# hiPSC-derived cardiomyocytes as a model to study the role of small-conductance Ca^2+^-activated K^+^ (SK) ion channel variants associated with atrial fibrillation

**DOI:** 10.3389/fcell.2024.1298007

**Published:** 2024-01-18

**Authors:** Hosna Babini, Verónica Jiménez-Sábado, Ekaterina Stogova, Alia Arslanova, Mariam Butt, Saif Dababneh, Parisa Asghari, Edwin D. W. Moore, Thomas W. Claydon, Nipavan Chiamvimonvat, Leif Hove-Madsen, Glen F. Tibbits

**Affiliations:** ^1^ Cellular and Regenerative Medicine Centre, BC Children’s Hospital Research Institute, Vancouver, BC, Canada; ^2^ Biomedical Physiology and Kinesiology, Simon Fraser University, Burnaby, BC, Canada; ^3^ IIB SANT PAU, and CIBERCV, Hospital de la Santa Creu i Sant Pau, Barcelona, Spain; ^4^ Department of Cellular and Physiological Sciences, Faculty of Medicine, University of British Columbia, Vancouver, BC, Canada; ^5^ University of California, Davis, Davis, CA, United States; ^6^ Instituto de Investigaciones Biomédicas de Barcelona (IIBB-CSIC), Barcelona, Spain; ^7^ Molecular Biology and Biochemistry, Simon Fraser University, Burnaby, BC, Canada; ^8^ School of Biomedical Engineering, University of British Columbia, Vancouver, BC, Canada

**Keywords:** small-conductance Ca^2+^-activated K^+^ channels, atrial fibrillation, calcium, cardiac action potential, single nucleotide polymorphisms, human induced pluripotent stem cells, cardiomyocytes

## Abstract

Atrial fibrillation (AF), the most common arrhythmia, has been associated with different electrophysiological, molecular, and structural alterations in atrial cardiomyocytes. Therefore, more studies are required to elucidate the genetic and molecular basis of AF. Various genome-wide association studies (GWAS) have strongly associated different single nucleotide polymorphisms (SNPs) with AF. One of these GWAS identified the rs13376333 risk SNP as the most significant one from the 1q21 chromosomal region. The rs13376333 risk SNP is intronic to the *KCNN3* gene that encodes for small conductance calcium-activated potassium channels type 3 (SK3). However, the functional electrophysiological effects of this variant are not known. SK channels represent a unique family of K^+^ channels, primarily regulated by cytosolic Ca^2+^ concentration, and different studies support their critical role in the regulation of atrial excitability and consequently in the development of arrhythmias like AF. Since different studies have shown that both upregulation and downregulation of SK3 channels can lead to arrhythmias by different mechanisms, an important goal is to elucidate whether the rs13376333 risk SNP is a gain-of-function (GoF) or a loss-of-function (LoF) variant. A better understanding of the functional consequences associated with these SNPs could influence clinical practice guidelines by improving genotype-based risk stratification and personalized treatment. Although research using native human atrial cardiomyocytes and animal models has provided useful insights, each model has its limitations. Therefore, there is a critical need to develop a human-derived model that represents human physiology more accurately than existing animal models. In this context, research with human induced pluripotent stem cells (hiPSC) and subsequent generation of cardiomyocytes derived from hiPSC (hiPSC-CMs) has revealed the underlying causes of various cardiovascular diseases and identified treatment opportunities that were not possible using *in vitro* or *in vivo* studies with animal models. Thus, the ability to generate atrial cardiomyocytes and atrial tissue derived from hiPSCs from human/patients with specific genetic diseases, incorporating novel genetic editing tools to generate isogenic controls and organelle-specific reporters, and 3D bioprinting of atrial tissue could be essential to study AF pathophysiological mechanisms. In this review, we will first give an overview of SK-channel function, its role in atrial fibrillation and outline pathophysiological mechanisms of *KCNN3* risk SNPs. We will then highlight the advantages of using the hiPSC-CM model to investigate SNPs associated with AF, while addressing limitations and best practices for rigorous hiPSC studies.

## 1 Introduction

Atrial fibrillation (AF) is the most common form of sustained arrhythmia globally, with a prevalence of approximately 5% in individuals younger than 60 years that could triple by the age of 80 (Heeringa et al., 2006; Zoni-Berisso et al., 2014; Bikdeli et al., 2022). Its presence is associated with a 5-fold increase in the stroke incidence rate, in addition to an increased mortality rate by 1.5-fold in men and 2-fold in women (Kraft et al., 2021). The lifetime risk of AF ranges from 23% to 38% depending on other risk factors (e.g., smoking, hypertension, myocardial infarction) (Staerk et al., 2018). AF is characterized by an ectopic rhythm originating in the atria. If the high frequency of excitation in AF remains untreated, it can result in severe consequences, including stroke and heart failure (HF) (Kamel et al., 2016; Xu et al., 2016; Gharanei et al., 2022). Nevertheless, despite its high prevalence in the community and its clinical importance, not all patients respond well to the current therapies.

Despite numerous studies investigating the electrophysiological, molecular, and structural alterations associated with AF, the genetic and molecular bases underlying AF pathogenesis and progression remain unclear. Familial AF cases can often be explained by point variants in genes that encode various ion channels (Mahida et al., 2011). Genome-wide association studies (GWAS) have additionally enabled the association of AF with myriad single nucleotide polymorphisms (SNPs) located in intronic or intergenic regions, including the 4q25, 1q21, and 16q22 chromosomal regions (Sinner et al., 2011; Liu et al., 2012). A recent study linked the risk SNP rs13143308T in the 4q25 chromosomal region with excessive Ca^2+^ release and spontaneous electrical activity in human atrial myocytes (Herraiz-Martínez et al., 2019). A better understanding of the functional consequences associated with these SNPs would allow for the use of specific SNP profiles as predictors of electrophysiological dysfunction. These data could influence clinical practice guidelines by improving genotype-based risk stratification and personalized treatment.

Although studies involving murine models have provided useful insights into the cause of AF, important differences in their ion channel expression profiles, intrinsic heart rates, and physiological regulatory mechanisms significantly hinder the translatability of data from these models to human cardiac function. For instance, the mouse heart rate is almost an order of magnitude higher than the human heart rate, and Phase 3 repolarization in rodents is mainly dependent on transient outward K^+^ current (I_to_) and slowly inactivating K^+^ currents (I_Kslow1/2_) (Kaese and Verheule, 2012). On the other hand, native human cardiomyocytes are challenging to obtain, incompatible with long-term culture, and cannot be readily genome-edited to examine their intricate molecular biology (Abi-Gerges et al., 2020).

Therefore, there is a critical need to develop a human-derived model that represents human physiology better than the existing animal models. In this context, the ability to generate atrial cardiomyocytes derived from human induced pluripotent stem cells (hiPSC-aCMs) from patients with specific genetic diseases, incorporating novel genetic editing tools to generate isogenic controls, organelle-specific reporters, and 3D bioprinting of atrial tissue could be transformative in improving our understanding of the mechanisms of AF and potential therapeutics. Looking forward, hiPSC-CMs are the most promising human source with the preserved genetic background of healthy donors or patients harboring variants thought to be associated with AF (Pioner et al., 2019). In this review, we highlight the advantages of using the hiPSC-CM model to investigate SNPs associated with AF while addressing limitations and best practices for rigorous hiPSC studies.

## 2 SK channel structure, function and regulation

A GWAS conducted by Ellinor et al. (2010) identified the rs13376333 risk SNP as the most significant one located in the 1q21 chromosomal region (Odds Ratio (OR) = 1.56) associated with AF. This variant is located within intron 1 of *KCNN3*, which encodes the small-conductance Ca^2+^-activated K^+^ channel type 3 (SK3). Since both upregulation and downregulation of SK channel expression can potentially promote arrhythmias, the current debate is whether this variant is a gain-of-function (GoF) or a loss-of-function (LoF) variant.

SK channels represent a unique family of K^+^ channels as they are voltage-independent channels that are primarily regulated by cytosolic Ca^2+^ ([Ca^2+^]_i_) thereby providing a link between changes in [Ca^2+^]_i_ and membrane potential (Adelman et al., 2012). This family consists of 3 ion channel paralogs (SK1-SK3), encoded by the genes *KCNN1-3* (Adelman et al., 2012; Zhang et al., 2015). All three paralogs have been identified in human and mouse hearts (Xu et al., 2003; Tuteja et al., 2005). However, some studies have demonstrated that the expression of SK channels in human atrial cells is higher than in human ventricular cells, and SK2 and SK3 expression in atrial cells is higher than SK1 expression (Skibsbye et al., 2014).

### 2.1 SK channel structure

Several studies have shed light on the structure of SK channels and their role in cardiac function (Zhang et al., 2015; Lee and MacKinnon, 2018). Like other K^+^ channels, the SK ion channel has six transmembrane segments (S1–S6), with a pore loop between S5 and S6. SK channels are voltage-independent, and their open probability is largely controlled by the local Ca^2+^ concentration. With physiological ion gradients, SK channels are unresponsive to changes in transmembrane voltage, because the S4 segment, which confers voltage-sensitivity in other channels, contains uncharged or less positively charged residues (Brown et al., 2022).

The co-expression of the different SK paralogs in a heterologous expression system can produce heteromeric channels (Tuteja et al., 2010). SK2 and SK3 subunits are known to form heterotetrameric channels, and both are co-localized within Z-lines in isolated atrial cardiomyocytes (Butler et al., 2023; Nam et al., 2023). SK2 and SK1 subtypes can form heterotetramer and heteromultimerization of SK1, SK2, and SK3 has also been reported (Benton et al., 2003; Monaghan et al., 2004). Thus, the study of the functional consequences of SK channels may be complicated by the multimerization of different sub-family members. Therefore, studying genetic variants in models such as hiPSC-CMs provides a more physiologically relevant complex system to evaluate phenotypic outcomes when compared with heterologous expression systems.

### 2.2 SK channel regulation

SK channels are activated by an increase in [Ca^2+^]_i_ within the atrial cardiomyocyte microdomain in which they exist, but the mechanism of SK channel activation by a rise in [Ca^2+^]_i_ is not well understood. It has been shown that calmodulin (CaM) is associated with SK channels through a CaM-binding domain (CaMBD) found in the C-terminus of the SK channel (Köhler et al., 1996; Xia et al., 1998). In this way, microdomain calcium binds to the CaM EF-hands, and the Ca^2+^-CaM complex binds the CaMBD of the SK channel, producing conformational changes that will activate the channel (Adelman et al., 2012; Zhang et al., 2018; Ledford et al., 2020). Based on cryogenic electron microscopic (cryo-EM) studies from the MacKinnon lab, under diastolic Ca^2+^ levels, the C lobe of CaM is constitutively bound to the CaMBDs on each of the four C-termini of the tetrameric channel and it remains in a closed state. As the Ca^2+^ levels rise, more Ca^2+^ binds to the N lobe of CaM allowing it to interact with the S4-S5 linker of the SK channel. The S4-S5 linker, which contains two distinct helices, undergoes conformational changes upon CaM binding to open the channel pore (Lee and MacKinnon, 2018).

Modelling the calcium regulation of SK ion channels has been of great interest. An elegant paper by Kennedy et al. (2017) studied the dynamic effects of Ca^2+^-sensitive potassium currents on voltage and calcium alternans, in which they developed a sophisticated model of the SK channel and integrated it with a physiologically detailed ionic model of a ventricular myocyte using the Ca^2+^ dependence formulation by Hirschberg et al. (1998). The governing equations for the modelling of SK ion channel currents are the following:

$$I_{\text{SK}}=g_{sk}{\;x}_{sk}\left(V_{m}-\;E_{K}\right)$$

$$x_{sk}=\frac{x_{sk\infty }-\;x_{sk}}{\tau_{sk}}$$

$$x_{sk\infty }=0.81\;\frac{c_{s}^{n}}{c_{s}^{n}-{EC}_{50}^{n}}$$

$$\tau_{sk}=\frac{1.0}{\left(0.047\;c_{s}+\frac{1}{76}\right)}$$

Using this model, they investigated the open probability (P_o_) of the SK channel by applying various test [Ca^2+^] pulses (0.2, 0.6, 1.0, and 1.8 μM). The results showed that the P_o_ of the SK channel increases as the [Ca^2+^] increases, reaching an open probability around 0.7 when the [Ca^2+^] is 1.8 μM. They also varied the EC_50_ from 0.1 to 1 μM to cover the whole range of physiological and pathophysiological conditions. The model showed that when Ca^2+^ affinity is high (lower EC_50_), the SK current (I_SK_) shortens both short and long action potentials (AP) regardless of the amplitude of the Ca^2+^ transient. However, when Ca^2+^ affinity is lower (higher EC_50_), the I_SK_ shortens the AP duration (APD) only when the amplitude of the Ca^2+^ transient is large. The challenging part of this modelling is that it is highly dependent on the microdomain in which the SK channels exist in atrial cardiomyocytes as outlined below, and that the equations used in this paper were derived from ventricular myocyte models.

### 2.3 The cardiomyocyte SK channel microdomain

**FIGURE 1:**
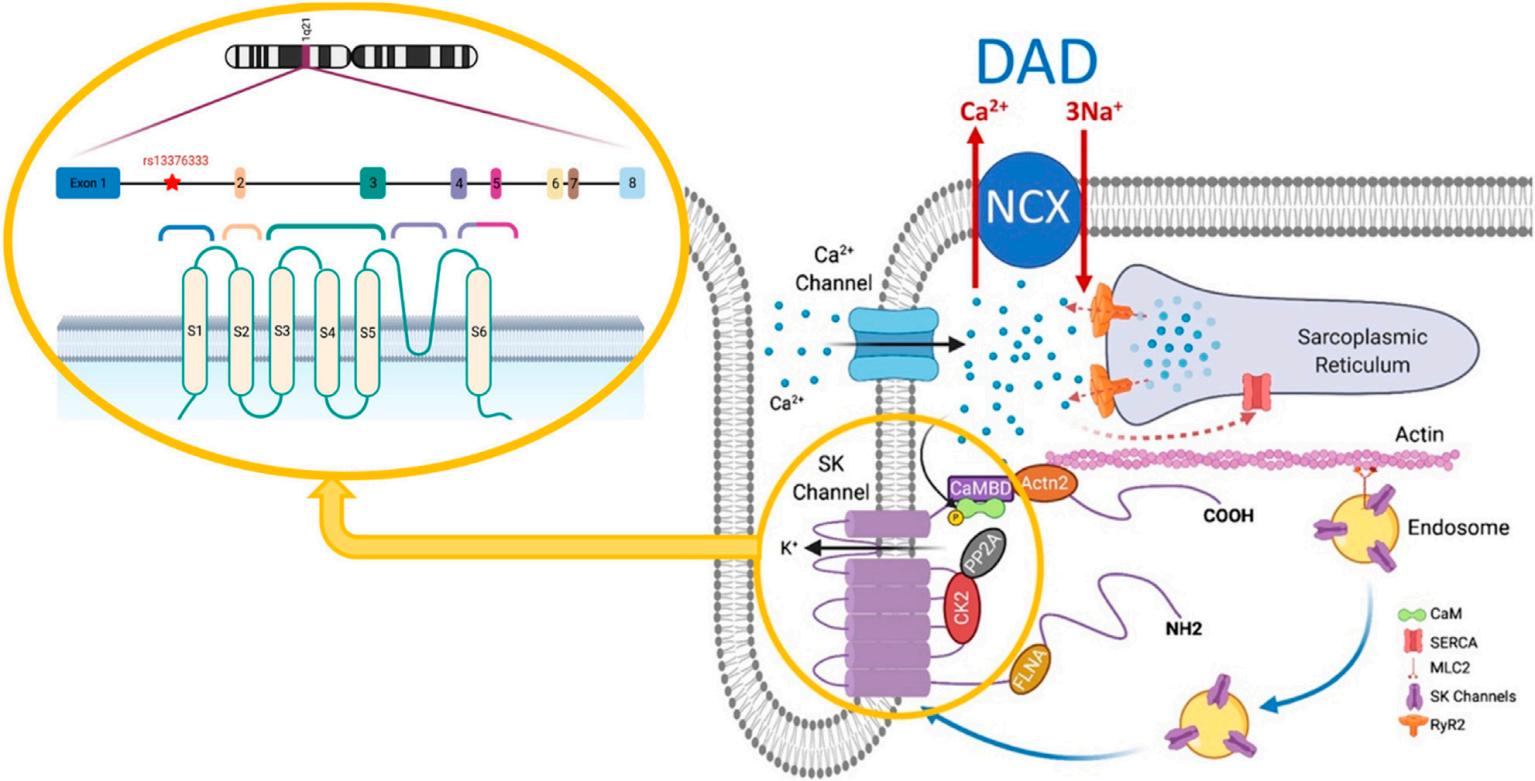
SK channel structure and interactome. Modified from Zhang et al. (2021), *Pflügers Archiv*, 473, 477. DAD, delayed afterdepolarization; NCX, Na^+^/Ca^2+^ exchanger; CaM, calmodulin; SERCA, sarcoplasmic/endoplasmic reticulum Ca^2+^-ATPase; CaMBD, calmodulin binding domain; MLC2, myosin light chain 2; FLNA, filamin A; CK2, casein kinase II; PP2A, protein phosphatase 2A; SK, small-conductance Ca^2+^-activated K^+^ channel; RyR2, cardiac ryanodine receptor.

The nature of the response of the SK channels to elevated Ca^2+^ levels is highly dependent on the physical properties of the microdomain, including its dimensions and the intrinsic Ca^2+^ buffering capacity in which they reside within the cardiomyocyte. It has been shown that SK channels form macromolecular complexes in which different proteins, such as CaM, α-actinin-2 (Lu et al., 2009), and filamin A (Rafizadeh et al., 2014), are postulated to interact with the channel as shown in Figure 1. These data strongly suggest that cardiac SK2 channels are functionally coupled to Ca_v_1.2 and Ca_v_1.3 ion channels through an EF-hand on α-actinin-2, which may explain their dependence on Ca_v_1.2 currents (Lu et al., 2009). Some studies have demonstrated that a reduction in α-actinin-2 or filamin A reduces SK2 expression (Lu et al., 2009; Rafizadeh et al., 2014). Studies in mice models have suggested that Ca^2+^ released by the cardiac ryanodine receptors (RyR2) embedded in the sarcoplasmic reticulum (SR) are necessary for the activation of SK channels (Mu et al., 2014; Terentyev et al., 2014). A recent study (Zhang et al., 2018) established the relation between the SK2 channels and Ca_v_1.2 and between SK2 channels and RyR2 using super-resolution images from rabbit ventricular cardiomyocytes. This suggests that both the entry of calcium through Ca_v_1.2 and the calcium released from SR by RyR2 produce an efficient calcium coupling to activate the SK channels in cardiomyocytes and that both mechanisms are important.

**FIGURE 2:**
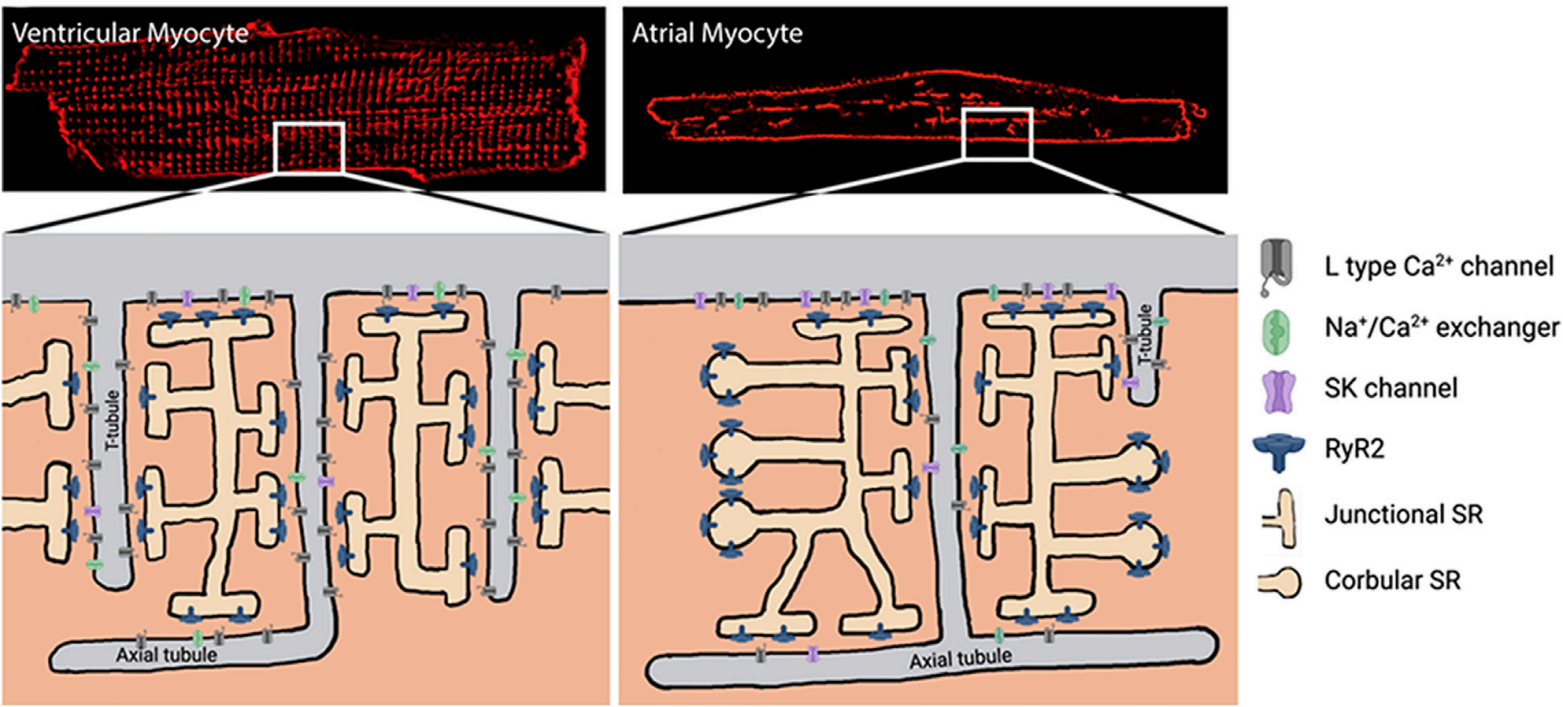
Dyad formation in ventricular vs. atrial cardiomyocytes. Modified from Schönleitner et al. (2017), *Progress in biophysics and molecular biology*, 130: 288-301. SK, small-conductance Ca^2+^-activated K^+^ channel; RyR2, cardiac ryanodine receptor; SR, sarcoplasmic reticulum.

As shown in Figure 1, the microdomain in which SK channels reside has been proposed to be part of the dyadic space where the T-tubules expressing Ca_v_1.2 and the junctional SR expressing RyR2 oppose each other. Thus, understanding the brief and controlled Ca^2+^ changes within the SK ion channel microdomain is crucial for the signaling properties of SK channels (Fakler and Adelman, 2008). However, this dyadic space microdomain concept in atrial cells is not as clear as it is for ventricular cells. In extensive work done on native atrial cells from murine hearts (Schulson et al., 2011), it was shown that the atrial T-tubular system is not as well developed as in ventricular cells. The atrial RyR2 complexes in rat atrial cells were found to be heterogeneous and several different types of RyR2 aggregates were identified based on their binding partners. One study argues that transverse tubules in the atrial cells of large mammals (e.g., sheep, horse, cow, and human) are more developed than those in murine models and play a more critical role in atrial excitation-contraction coupling (Richards et al., 2011). However, several studies in native human right atrial cardiomyocytes have shown that the development of T-tubules is very low in comparison to human ventricular cardiomyocytes (Llach et al., 2011a; Tarifa et al., 2023). Moreover, another study in native human right atrial cardiomyocytes demonstrated that the calcium transient propagates from the sarcolemma toward the cell center, which is in line with the low density of T-tubules (Herraiz-Martínez et al., 2015). A proposed model of atrial dyads is shown below in Figure 2, in which the heterogeneity of the dyads adds a significant degree of complexity (Schönleitner et al., 2017). Therefore, a better understanding of the microdomain environment of SK ion channels will provide new perspectives on Ca^2+^ signaling networks (Fakler and Adelman, 2008).

### 2.4 SK channel trafficking

Another critical aspect that impacts SK channel current density is the trafficking of the channels from the endoplasmic reticulum to the sarcolemma. Studies have shown that Ca^2+^-independent CaM binding to the SK channel is necessary to express SK channels in the membrane surface (Zhang et al., 2021), and several studies have shown that SK channel trafficking is a Ca^2+^-dependent mechanism. Rafizadeh et al. (2014) showed that a significant reduction of SK2 channels membrane localization was associated with a decrease in [Ca^2+^]_i_-dependent repolarization. On the other hand, a study in isolated rabbit atria demonstrated a significant increase in SK2 channel density in the plasma membrane in the pulmonary vein region after burst pacing (Ozgen et al., 2007). These results suggest that an increase in [Ca^2+^]_i_ due to rapid pacing could increase the forward trafficking of SK2 channels. A recent study also showed a physical relationship between junctophilin type 2 (JP2) and SK2 channels in cardiomyocytes (Fan et al., 2018). Thus, altering the expression or activity of any of the proteins that interact with SK channels could regulate their expression and function (Lu et al., 2009; Lu et al., 2015; Rafizadeh et al., 2014). Moreover, several studies have associated the alteration in some of these proteins, such as JP2, Ca_v_1.2, and Ca_v_1.3, with AF (Llach et al., 2011a; Beavers et al., 2013; Greiser and Schotten, 2013; Sun et al., 2017), suggesting the importance of studying the proteins that interact with SK channels to understand the role of SK channels in AF.

It is clear that there are numerous gaps in our knowledge base. Further research is required to elucidate the differential expression of the different paralogs of the SK channels in atria and ventricle, the functional role of SK channels in pacemaking cells and the cardiac conduction system, the SK channel expression and function in intracellular organelles such as mitochondria (Kim et al., 2017), and the interactome of SK channels at a microdomain level.

## 3 SK channels and AF

Studies of SK channels strongly support their critical role in the regulation of atrial excitability (Zhang et al., 2021). One of the principal mechanisms associated with AF is electrical ectopic *foci* in the pulmonary veins and electrical re-entry circuits (Wit and Boyden, 2007). In addition, the structural remodelling involved in atrial fibrosis or atrial dilatation has been linked to the initiation of AF (Velagapudi et al., 2013; Corradi, 2014). At a molecular level, AF has been associated with different alterations in Ca^2+^ homeostasis, including 1) reduction in the L-type calcium current (Van Wagoner et al., 1999; Llach et al., 2011a; Greiser and Schotten, 2013); 2) an increase in spontaneous calcium release from the SR (Hove-Madsen et al., 2004; Llach et al., 2011b), and 3) an increase in delayed afterdepolarizations (DADs) induced by spontaneous calcium release (Voigt et al., 2012; Tarifa et al., 2023). The electrical remodelling caused by the shortening of the atrial refractory period and APD have also been proposed as mechanisms for AF development (Pang et al., 2011). Current therapies for AF mainly include drug therapy (e.g., β adrenergic receptor (βAR) blockers) and catheter ablation (Nattel et al., 2021). A recent study suggested that catheter ablation may be more effective as a first-line therapy (Andrade et al., 2021). However, every approach has its limitations, and further investigation is necessary to obtain optimal treatment for each patient with AF.

The possible role of SK channels in AF pathogenesis is complex and controversial. Several studies have associated AF with increased SK channel expression and function in different models. A recent paper (Heijman et al., 2023) supports the concept that overexpression of SK channels in native human atrial cardiomyocytes is causal for AF. They also found that SK2 channel forward trafficking was Ca^2+^ and α-actinin-dependent in AF. Additional data in mice support the idea that overexpressing SK3 channels would considerably shorten atrial APD, mostly by lowering APD_90_ compared to APD_50_. At the same time, knock-out (KO) of SK3 channels had the reverse effect on APD (Zhang et al., 2021). Overexpression of the SK3 channels has been linked to the pro-fibrillatory effects in the atrium (Mahida et al., 2014), and pacing-induced AF was treated with SK channel inhibitors to treat arrhythmias in several trials utilizing animal models (e.g., rat, mouse, and guinea pig) (Bentzen et al., 2020). In these circumstances, SK2 or SK3 inhibitors may be an effective alternative for treating AF.

On the contrary, other studies have associated AF with a reduction in SK channel expression and function. One reported that individuals with persistent AF have decreased SK2 and SK3 expression compared to control patients in sinus rhythm (Skibsbye et al., 2014). Another study showed that patients with persistent AF had lower SK current levels than those in sinus rhythm (Yu et al., 2012).

Although research using native human atrial myocytes is attractive because it facilitates clinically relevant translation, the variability and interpretation can be complicated by multiple factors such as patient co-morbidities (Molina et al., 2018), chronic pharmacological intervention (Jiménez-Sábado et al., 2023), remodeling (Dinanian et al., 2008; Molina et al., 2018), variable genetic backgrounds, sex (Herraiz-Martínez et al., 2022), age (Herraiz-Martínez et al., 2015), anatomic region (Qi et al., 2014), and presence of specific systemic modulators (Rahm et al., 2021), among others.

### 3.1 Pathophysiological mechanisms of SK risk SNPs associated with AF

GWAS-derived data link common SNPs in *KCNN2* and *KCNN3* with AF (Ellinor et al., 2010; Roselli et al., 2018). The rs13376333 risk SNP located within intron 1 of the *KCNN3* gene seems to be the most significant one, with an OR of 1.56 (Ellinor et al., 2010). In this context, a recent study has linked the rs13376333 variant to an increased *KCNN3* mRNA-expression in human atria (Bentzen et al., 2020). However, since it has been suggested that SK ion channel trafficking is Ca^2+^ dependent (Ozgen et al., 2007; Rafizadeh et al., 2014; Heijman et al., 2023), mRNA levels by themselves may not be a reliable indicator of SK channel function.

**FIGURE 3:**
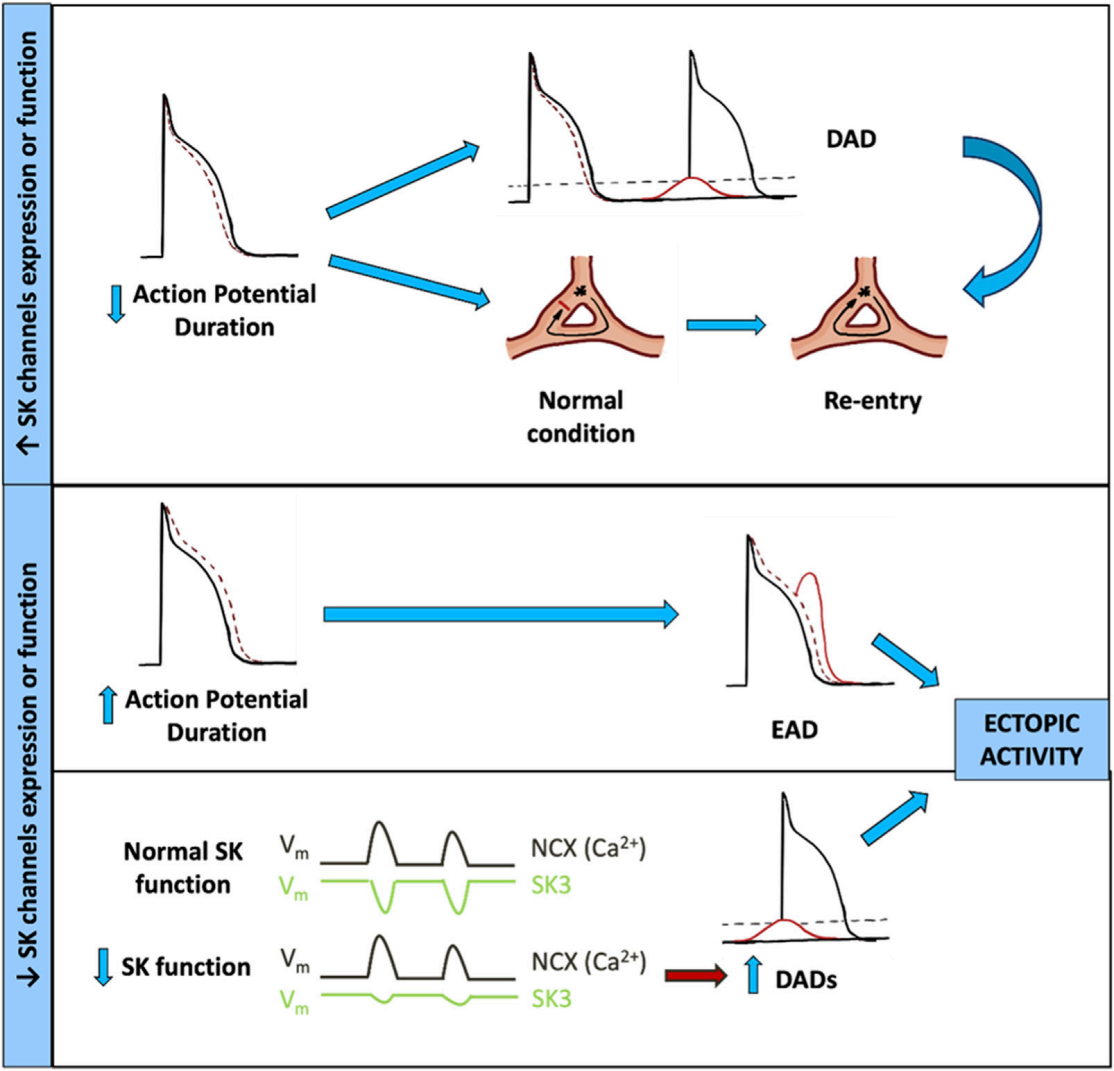
Contribution of SK channel dysfunction to AF arrhythmogenic mechanisms. DAD, delayed afterdepolarization; EAD, early afterdepolarization; NCX, Na^+^/Ca^2+^ exchanger; SK3, small-conductance Ca^2+^-activated K^+^ channel type 3.

Thus, more studies are required to elucidate whether the rs13376333 risk variant from the *KCNN3* gene is a GoF or LoF variant because both potentially promote arrhythmias but would likely require different interventions. The pathophysiological mechanisms in which the downregulation or upregulation of SK channels could be involved are summarized in Figure 3.

In the GoF hypothesis, the increase in *KCNN3* expression and function would produce an increase of SK3 current, resulting in a shortening of the effective refractory period (ERP) and APD due to a faster repolarization of the AP. The shortening of ERP can facilitate DAD generation and the more likely propagation of an aberrant AP, increasing the risk of re-entry mechanism and arrhythmia.

Alternatively, in the LoF hypothesis, at least two different mechanisms could be involved in the pathogenesis of AF. First, the reduction in the expression and function of SK3 channels would result in a reduction of the SK3 current density, prolonging the repolarization phase and consequently prolonging both the ERP and APD. This increase in APD would facilitate EADs, conduction heterogeneity, or conduction block if the APD increase is significant. Another LoF hypothesis suggests that by reducing the expression and function of the SK3 channel, there will be less outward current to counteract the depolarizations generated by NCX1 in response to RyR2 Ca^2+^ release, thereby increasing DAD magnitude. Both mechanisms could produce AF by the induction of ectopic activity *foci.*

## 4 hiPSC-CMs as a model for the study of SK channel risk variants

Research with hiPSCs and the subsequent generation of hiPSC-CMs has proven to be transformative in cardiovascular research. hiPSC-CMs have been widely used to model various genetic disorders (Itzhaki et al., 2011; Shafaattalab et al., 2019a; Shafaattalab et al., 2021; Arslanova et al., 2021; Hong et al., 2021; Barc et al., 2022; Kondo et al., 2022), screen for efficacy and safety during drug development, and investigate mechanisms of cardiotoxicity (Christidi et al., 2020; Huang et al., 2022). This technology allows pluripotent stem cells to be derived from healthy donors and patients harboring different genetic variants. hiPSC-CM models have revealed the underlying causes of various diseases and identified treatment opportunities that were not possible using *in vitro* or *in vivo* studies with animal models.

A number of studies have shown that hiPSC-CMs can be successfully used as a model for investigations of channelopathies such as long QT syndrome (LQTS) (Moretti et al., 2010; Venkateshappa et al., 2023), short QT (SQT) (El-Battrawy et al., 2018), Brugada syndrome (BrS) (Liang et al., 2016), and Catecholaminergic Polymorphic Ventricular Tachycardia (CPVT) (Zhang et al., 2013; Park et al., 2019; Tani and Tohyama, 2022; Kim et al., 2023). Moreover, a recent study in hiPSC-aCMs has shown that AF-associated electrical remodeling can be induced in hiPSC-aCMs, suggesting that this model can be used to study the underlying mechanisms of AF (Seibertz et al., 2023).

In addition, electrophysiological and transcriptomic studies have demonstrated that hiPSC-CMs express SK channels (Zhao et al., 2018; Simons et al., 2023). However, the expression of SK channels in hiPSC-aCMs has not been studied. If done rigorously, hiPSC-CMs can provide a powerful platform for investigating the effect of SK3 channels on APD and electrical disturbances associated with AF. Being an intronic variant, it makes the hiPSC-aCM model particularly insightful as this cannot be done in heterologous expression systems. If the 1q21 variant proves to be significant in causing AF, the SK3 channels may be a novel target for pharmaceutical intervention, and the hiPSC-CM model can be used for assessing the efficacy and toxicity of novel antiarrhythmic drugs (i.e., SK channel blockers/activators).

However, to obtain a powerful hiPSC model for studying human diseases, it is important to establish consistent standards and best practices for tissue procurement, hiPSC reprogramming, day-to-day cultivation, quality control, and data management aligned with an ethical and legal framework (Allsopp et al., 2019; Steeg et al., 2021). In this context, genomic integrity plays a fundamental role in the functional and therapeutic potential of hiPSC models. Aberrations or abnormalities in the genomic structure of hiPSCs could induce undesired consequences, including karyotyping abnormalities, single nucleotide variations (SNVs), copy number variations (CNVs), loss of heterozygosity, increased risk of tumorigenesis, or compromised functionality of differentiated cells (Mayshar et al., 2010; Martins-Taylor and Xu, 2012; Araki et al., 2020; Kanchan et al., 2020). It has been documented that such abnormalities may arise through three distinct pathways: i) hiPSCs inheriting pre-existing variations present in the donor cells, ii) mutations being introduced by the reprogramming process itself, and iii) prolonged/long-term hiPSC culturing through passage iterations. This section describes the standards in place to ensure high-quality hiPSC approaches that are applicable to studying SK channel variants, as well as more broadly.

### 4.1 Reprogramming patient somatic cells to hiPSCs

hiPSCs can be generated from different somatic cells such as blood, skin, and others (Aasen et al., 2008; Kim et al., 2009; Loh et al., 2009). Among those, non-invasive methods for tissue sampling are preferable. This process is usually performed using four reprogramming transcription factors generally referred to as the Yamanaka factors: SOX2 (sex-determining region Y-box 2), Oct3/4 (Octamer-binding protein 4), Klf4 (Krüppel-like factor 4), and c-Myc. These Yamanaka factors can be delivered to the somatic cell using a non-integrating viral construct (e.g., Sendai virus) to transform the somatic cells into hiPSCs (Hu and Zhang, 2010).

Thus, the reprogramming process itself should be carefully optimized to minimize the introduction of genetic aberrations. To date, the vastly utilized reprogramming approaches include Sendai-viral (SeV) (Fusaki et al., 2009), Lenti- and Retro-viral (Park et al., 2008; Somers et al., 2010), episomal (Okita et al., 2011) and mRNA (Warren et al., 2010) transfection methods. A comparison of these reprogramming approaches has shown notable variations in aneuploidy rates, reprogramming efficiency, reliability, and the workload of generating hiPSCs (Schlaeger et al., 2015; Steichen et al., 2019). Using non-integrating reprogramming methods, such as mRNA or protein-based reprogramming factors, can also help reduce the risk of genomic instability.

### 4.2 Genome editing

An hiPSC line can be used as a model of known genomic background into which a desired risk variant can be incorporated, and its effects studied through functional analyses. Moreover, the same risk variant can be introduced into multiple cell lines from different sex and genomic backgrounds to examine the sex-dependent and genetic background-dependent differences. This reduces the limitation of any confounding genetic abnormalities and allows direct assessment to characterize cells from genotype to phenotype. Many challenges are faced when comparing hiPSC-CMs from a patient harboring a genetic variant of interest to those derived from a “healthy control” donor. The interpretation of the impact of the variant is far more rigorous if one genome edits the patient’s hiPSC line to “reverse” the variant and compares the variant to its isogenic control.

In the case of studying AF, commercially acquired hiPSCs and patient-derived hiPSCs can be used as relevant disease models (Hong et al., 2021).

### 4.3 Differentiation of hiPSCs into atrial and ventricular cardiomyocytes

**FIGURE 4:**
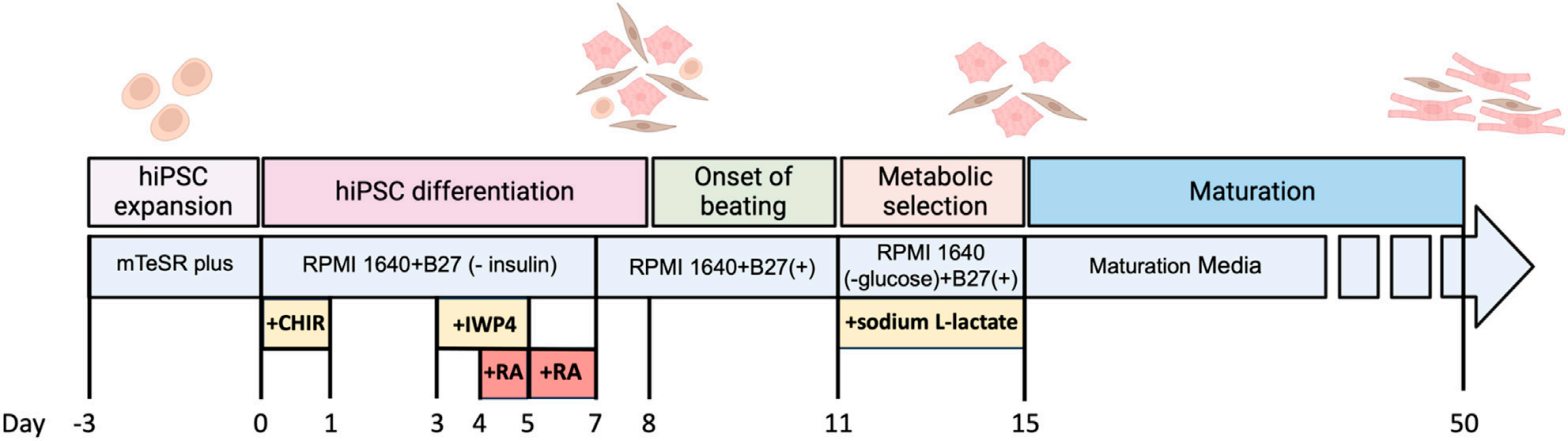
Timeline for differentiation of hiPSCs to atrial cardiomyocytes and the maturation process.

Figure 4 shows optimized protocols that have been developed in our lab to differentiate the hiPSCs cell lines harbouring the SK risk variants and their isogenic control into either atrial cardiomyocytes (aCMs) or ventricular cardiomyocytes (vCMs) (Lian et al., 2013; Gunawan et al., 2021). The main difference in differentiating hiPSCs into atrial cardiomyocytes is the addition of 0.75 µM retinoic acid (RA) from days 4–6 (Shafaattalab et al., 2019b; Gharanei et al., 2022). The exposure to RA in this narrow developmental window converts a sub-population of cardiovascular mesoderm cells that contribute specifically to the posterior regions of the cardiac crescent and gives rise to the atria CMs (Devalla et al., 2015; Lee et al., 2017; Protze et al., 2019). These differentiated CMs can be put through either metabolic selection medium or magnetic-activated cell sorting (MACS) to purify them from non-cardiomyocytes prior to morphologic, transcriptomic, proteomic, and electrophysiological characterization (Sharma et al., 2015).

Thus, the use of hiPSC-CMs can allow one to study not only the phenotypic outcome of an SK channel genetic risk variant, but also the influence on expression differences of SK channels between ventricular and atrial cardiomyocytes, which is one of the gaps that need to be addressed.

### 4.4 Ion channel expression profiles and maturation

hiPSC-CMs express most ion channels required for the cardiac AP. However, one major limitation of hiPSC-CMs has been their relative immaturity compared to primary adult cardiomyocytes, which is reflected in their electrophysiological and Ca^2+^ handling properties. Immature hiPSC-CMs display lower Phase 0 upstroke velocity owing to lower expression of functional Na_V_1.5 channels (Ma et al., 2011; Zhao et al., 2018). Additionally, immature hiPSC-CMs have a relatively more depolarized resting membrane potential (RMP) as a result of lower K_ir_2.1-encoded I_K1_ and higher *HCN4*-encoded I_f_ current densities (Satin et al., 2004; Zhao et al., 2018). This also contributes to the spontaneous beating of immature hiPSC-CMs, which is not a feature of native adult cardiomyocytes. Slightly lower expression of functional I_to_-encoding channels, as well as a relatively higher K_V_1.4 (I_to,s_) and lower KChIP2 (I_to,f_) expression, can lead to a relatively smaller contribution of I_to_ to repolarization in immature hiPSC-CMs (Ma et al., 2011; Cordeiro et al., 2013; Protze et al., 2017). In hiPSC-aCMs derived from RA-guided differentiation, I_Kur_ is relatively smaller than adult atrial cells but was sufficient to produce a shorter APD compared to hiPSC-vCMs (Hilderink et al., 2020). Otherwise, I_Ca,L_, I_Kr_, and I_Ks_ current densities and the expression of their encoding channels are considered comparable to adult cardiomyocytes (Itzhaki et al., 2011; Ma et al., 2011; Lahti et al., 2012), and I_KAch_ was recently shown to be comparable in hiPSC-aCMs (Ahmad et al., 2023).

The capacity for Ca^2+^ handling and storage in immature hiPSC-CMs is present but relatively incomplete, owing to the absence of well-developed T-tubule networks and lower expression of important Ca^2+^-related proteins such as RyR2, SERCA, and calsequestrin (CASQ2) (Kermani et al., 2023). This aspect is important to consider when investigating SK currents, which are present in hiPSC-CMs (Zhao et al., 2018), given their spatiotemporal relationship with Ca^2+^ sources for activation. To overcome limitations related to hiPSC-CM immaturity, tremendous efforts have gone into maturing hiPSC-CMs to improve their electro-contractile profiles, including long-term culture (Lundy et al., 2013; Lewandowski et al., 2018), biophysical modification such as extracellular matrices (Herron et al., 2016; Ogasawara et al., 2017) and polydimethylsiloxane (PDMS)-based polymers (Thavandiran et al., 2013) with low tensile strength (McCain et al., 2014; Ribeiro et al., 2015), metabolic and hormonal supplementation (Nakano et al., 2017; Parikh et al., 2017; Horikoshi et al., 2019; Yang et al., 2019; Feyen et al., 2020), electrical stimulation (Chan et al., 2013), and 3D tissue engineering (Huethorst et al., 2016; Lemoine et al., 2017; Correia et al., 2018; Ulmer et al., 2018; Silbernagel et al., 2020), which have been previously reviewed in more detail elsewhere (Hamledari et al., 2022). Importantly, many of these approaches have successfully improved the electro-contractile properties, as shown by: a) increased *KCNJ2* expression and I_K1_ current density along with lower RMP values (Nunes et al., 2013; Herron et al., 2016; Abilez et al., 2018; Horváth et al., 2018; Feyen et al., 2020; Huang et al., 2020), b) higher expression of *SCN5A* and upstroke velocity (Herron et al., 2016; Lemoine et al., 2017; Feyen et al., 2020), c) lower *HCN4* expression and I_f_ current density (Ronaldson-Bouchard et al., 2018) with lower intrinsic beating rates, d) greater Ca^2+^ handling, storage, and expression of associated proteins (RyR2, SERCA, NCX1, CASQ2) (Richards et al., 2016; Ruan et al., 2016; Parikh et al., 2017; Shadrin et al., 2017; Ronaldson-Bouchard et al., 2018; Feyen et al., 2020; Huang et al., 2020), and e) T-tubule formation and improved sarcomere alignment (Mills et al., 2017; Parikh et al., 2017; Ronaldson-Bouchard et al., 2018; Feyen et al., 2020; Huang et al., 2020). Although none of these methods can yet fully recapitulate years of *in vivo* maturation, their utilization drastically improves hiPSC-CMs as a translational model to study adult heart disorders. Thus, when feasible, applying validated maturation methodologies in hiPSC-CM studies is highly recommended.

### 4.5 Downstream analysis in hiPSCs-derived cardiomyocytes

As described above, one of the key advantages of the hiPSC-CM model for studying SK channel variants is the ability to determine the expression differences of SK channels between hiPSC-aCMs and hiPSC-vCMs harbouring SK risk variants and their isogenic controls. To do this, either qRT-PCR or a custom NanoString codeset can be used. Additionally, immunocytochemistry can be used to examine the co-localization of SK channels with RyR2, Ca_v_1.2, α-actinin-2 and other proteins speculated to be within the SK channel interactome in both hiPSC-aCMs and hiPSC-vCMs. Deconvolution and determining the degree of protein colocalization can be performed as described (Sedarat et al., 2004). The images can also be analyzed with a MatLab algorithm designed to determine the cluster density and the intensity ratio of the different proteins (Nolla-Colomer et al., 2022).

Functional validation of hiPSC-aCMs is crucial to ensure the differentiation capacity and can be evaluated using an array of techniques. Patch clamp can be used to assess the impact of the variant on the recorded current and spontaneous electrical activity in hiPSC-aCMs, and multiple electrode array (MEA) to determine the impact of the variant on electrical activity and stability. High-speed optical mapping (OM) can measure voltage, cytosolic Ca^2+^ transients and conduction velocity, and μOM can record Ca^2+^ sparks and waves. Finally, cleavage under targeted accessible chromatin (CUTAC) can be used to determine the transcriptional impact of the variant, and flow cytometry enables the study of protein expression.

## 5 Conclusions and future perspectives

Several studies over the past two decades have provided new insights into the role of SK channels in the regulation and function of cardiac excitability, suggesting that SK channels can be a new therapeutic approach for the treatment of cardiac arrhythmias. However, as we mentioned in this review, there are different knowledge gaps that still need more research.

In this context, the use of hiPSC-aCMs as a model to study the SK variants addresses some of the challenges found in different animal models and native human atrial cardiomyocytes. Following the practices described above, the hiPSC-CMs model can be a very powerful tool not only to study the underlying mechanisms of these genetic risk variants associated with AF but also to test different therapeutic approaches. In conclusion, the hiPSC-CMs model presents different advantages over other models in terms of the transnationality of the results to clinical practice.
